# Production of Alginate Oligosaccharides (AOSs) Using Enhanced Physicochemical Properties of Immobilized Alginate Lyase for Industrial Application

**DOI:** 10.3390/md22030120

**Published:** 2024-03-04

**Authors:** Simranjeet Kaur, Reinu E. Abraham, Christopher M. M. Franco, Munish Puri

**Affiliations:** Medical Biotechnology, College of Medicine and Public Health, Flinders University, Bedford Park, Adelaide, SA 5042, Australia

**Keywords:** immobilization, alginate lyase, magnetic nanoparticle, alginate oligosaccharides (AOSs), nanomaterial

## Abstract

Alginate lyase (AL) is a polysaccharide-degrading enzyme that can degrade alginate by hydrolyzing glycosidic bonds and produces unsaturated alginate oligosaccharides (AOSs). These AOSs have wide therapeutic and nutraceutical applications. However, to produce alginate oligosaccharides in a cost-effective manner is challenging due to the low availability and high cost of this degrading enzyme. Immobilization of the enzyme facilitates industrial applications owing to its stability, reusability, and cost-effectiveness. This study was focused on the enhancement of the properties of alginate lyase and improvement of the production of AOS. Alginate lyase was immobilized on magnetic nanoparticles (NPs) using glutaraldehyde as the crosslinker. The study showed that the maximum binding achieved between NPs and protein in the enzyme was 71% at a ratio of 1:150 NP:protein. As a result of immobilization, the optimum activity of free enzyme which was obtained at 37 °C and pH 7.4 changed to 45 °C and pH 9. Furthermore, the enzyme was thermostable at 45 °C for 3 h with up to 50% reusability for six consecutive cycles. Storage stability after 15 days showed ~67% relative hydrolysis of alginate. The free alginate lyase (25 IU) showed 76% raw biomass (seaweed) hydrolysis which is higher compared to 63% provided by the immobilized enzyme. As a result of efficient hydrolysis, AOSs with molecular weight profile of 370–1040 kDa were produced and detected using HPLC.

## 1. Introduction

Alginate lyase (AL) is a polysaccharide-degrading enzyme which follows a β-elimination mechanism during the hydrolysis of a hetero-polymer alginate [1]. Alginate lyase can be obtained from naturally occurring bacteria, algae, and echinoderms in the marine environment [2]. The spectacular physicochemical properties of alginate including its mechanical stiffness, ability to form hydrogels, and biocompatibility properties make it an ideal candidate for therapeutic and pharmaceutical application [3] and emulsifier and gelling agent for the food and beverage industry [4]. Alginate-derived products show bacteriostatic, antioxidant, antitumor, and immunomodulation properties and prevention of dental caries [5]. However, the high viscosity of the alginate limits its commercial application, therefore there is a significant demand for low-viscosity and low-molecular-weight alginate oligosaccharides (AOSs) in various industries [6]. The global market value of alginate derivatives is expected to increase from USD 442.6 million (2020) to USD 547.2 million (2027). Drugs based on alginate oligomers have been approved since 2019 in China to cure cystic fibrosis, Alzheimer’s disease, and neurological and metabolic diseases [7]. 

Enzymatic degradation of alginate into oligosaccharides is a safer and greener approach compared to physical and chemical hydrolysis due to less energy consumption and limited exposure to harmful chemicals [5]. The high cost of alginate lyase is a major challenge which limits its application. To address this challenge, this study aimed to immobilize alginate lyase to make it reusable and achieve an efficient hydrolysis [1]. Immobilization tailors and enhances the physicochemical properties of the enzyme by improving its enzymological characteristics such as catalytic activity, specificity, selectivity, and stability in a wide range of pH, temperatures, and solvents which make it efficient and reliable [8]. Using immobilized enzyme for the alginate hydrolysis is expected to be a cost-effective approach to fulfil the emerging demand for functional carbohydrates and oligosaccharides [9]. In previous studies, a number of natural, inorganic, and synthetic polymeric materials were used as supports to immobilize the enzymes. These compounds, such as cellulose [10], chitosan [11,12,13], and agarose, possess ideal support properties such as hydrophilicity, physical resistance to compression, resistance to microbial attacks, biocompatibility, mechanical stability, large surface area, and low-cost availability. However, there are limitations due to diffusion when macromolecular substrates are used as a carrier. The activity of enzymes such as pectin lyase is reported to be negatively affected by immobilization due to steric hindrance of the substrate to reach the active site of the enzyme [14]. Nanoparticles [15] are the ideal candidates to deal with this as they show Brownian movement which provides comparatively better enzymatic activity due to dispersion in aqueous solutions. Additionally, nanoparticles facilitate the easy separation of the bound enzyme from reaction mixture by using an external magnetic field [1,16]. Also, improved stability and enzymatic activity have been demonstrated by the reduction in protein unfolding due to immobilization. Biocatalytic efficiency, specific surface area, mass transfer resistance, and effective enzyme loading are the ideal characteristics of nanoparticle-based immobilization [17]. 

The aim of this study was to immobilize alginate lyase and produce alginate oligosaccharides (AOSs). The immobilization was confirmed by investigating its binding efficiency and characterizing its physiochemical properties. This study is a maiden attempt to employ immobilized alginate lyase for hydrolysis of raw macroalgal (seaweed) biomass to produce alginate oligosaccharides (AOSs). 

## 2. Results and Discussion

### 2.1. Effect of Metal Ions on Enzymatic Activity and Binding Efficiency of Immobilization

**Figure 1:**
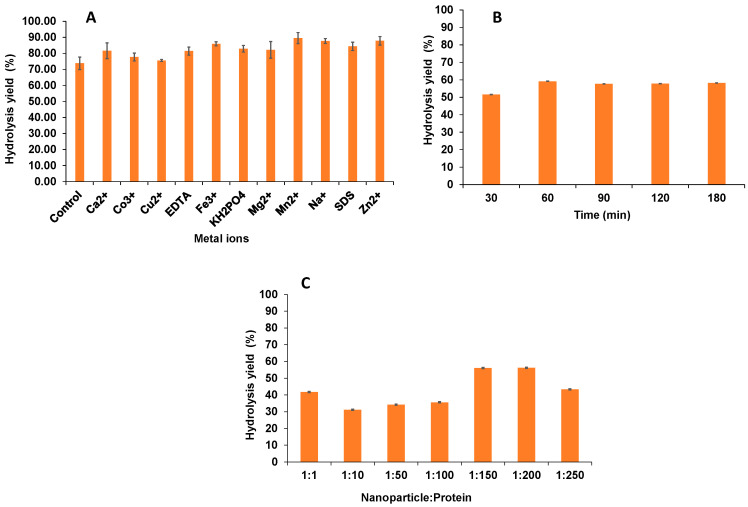
(**A**) Effect of different metal ions on the enzymatic activity of alginate lyase, (**B**) Activation of nanoparticles as a function of variable time, (**C**) Binding efficiency of alginate lyase onto magnetic nanoparticles with varying concentration of protein loaded.

Overall, the results showed that the addition of metal ions enhanced alginate lyase activity. Mn^2+^, Zn^2+^, Na^+^, and Fe^3+^ resulted in 89.5 ± 3.4%, 87.8 ± 2.6%, 87.7 ± 1.5%, and 85.9 ± 1.2% of the activity of the untreated samples, respectively, and enhanced the hydrolysis of alginate to more than 85% (~15% higher than control) (Figure 1A). However, the addition of Co^3+^ and Cu^2+^ showed 77.72% and 75.60% enzyme activity, respectively, which was slightly higher than that of the control (73.82%). Earlier studies have reported that different metal ions exhibit different effects on the activity of alginate lyase as observed in this study [5,18,19]. Previous findings reported the inhibitory effect of most of the metals including Na^+^ and Mg^2+^ which is contrary to the present findings [20]. The results from this study confirmed the enhanced activity of the enzyme by using magnetic nanoparticles for immobilization. Glutaraldehyde was used as the crosslinker. The maximum binding of glutaraldehyde with the nanoparticles was achieved within 1 h of incubation as shown in Figure 1B. About 50% relative activity was recorded in 30 min, and a further 9% increase was observed by doubling the time. There was no considerable improvement observed upon further extending the time to 180 min. As compared to other hydrolytic enzymes, pectinase was incubated overnight to achieve activation with glutaraldehyde (10% *v*/*v* of glutaraldehyde and 25 °C) [14] and, with chitosan nanoparticles, the activation of magnetic nanoparticles was achieved in a 30 min incubation [14]. However, this study demonstrated the maximum activation of the nanoparticles with the glutaraldehyde crosslinker was achieved in 60 min for immobilized AL. Hence, the time required for the activation of magnetic nanoparticles varies as compared to previous studies, based on the concentration and type of particle used. The binding efficiency of the protein load onto the magnetic nanoparticles was confirmed by protein assay. The protein loading was optimized by loading different concentrations of alginate lyase (units) onto nanoparticles and the results were analyzed by determining the enzymatic activity for various nanoparticle:protein ratios including 1:1, 1:10, 1:50, 1:100, 1:150, 1:200, 1:250 as shown in Figure 1C. There was a significant enhancement in the enzymatic activity with the increase in the protein:nanoparticle ratio. Upon increasing protein load from 1:10 to 1:50 and 1:100, a constant increase in the binding efficiency by 13% was observed. A maximum of 71% protein binding was observed at a protein:nanoparticle ratio of 1:150 (5 mg/mL of nanoparticles:750 mg of protein). With the further increment in the protein load to 1:250, no improvement in binding was observed (Figure 1C). No similar data for binding efficiency of alginate have been reported in the literature.

### 2.2. Characterization Studies

**Figure 2:**
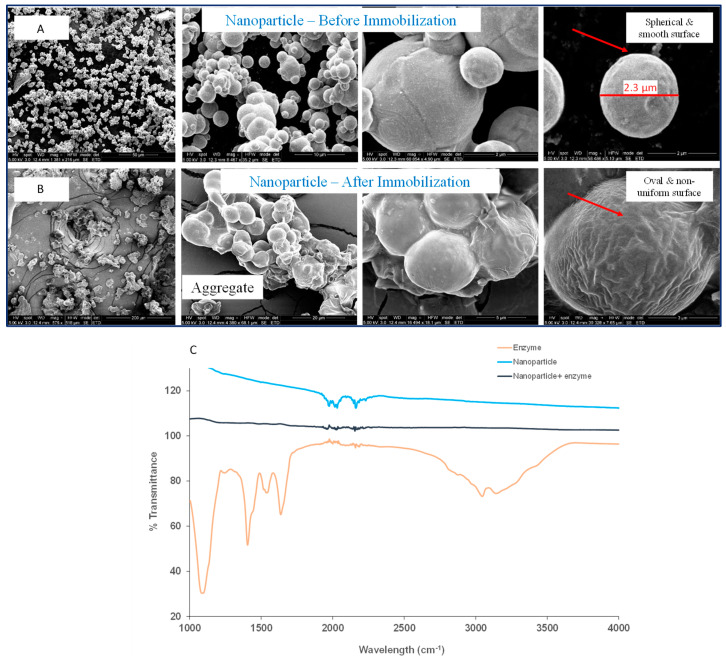
(**A**) SEM images for nanoparticles and (**B**) alginate lyase immobilized on nanoparticles at an accelerating voltage of 5 kV and magnification ranges from 500 nm to 300 µm; (**C**) FTIR spectrum of magnetic nanoparticles, soluble alginate lyase, immobilized alginate lyase.

The immobilized enzyme was characterized using scanning electron microscopy (SEM) as shown in Figure 2A,B. The study shows that the nanoparticles were spherical in shape, with size ranging from 500 nm to 300 µm. Before immobilization, the nanoparticle structure appeared to be smooth, spherical, uniform, and spongy. However, after immobilization the size of the nanoparticles appeared to be enlarged and they formed aggregated clusters as shown in Figure 2B. This suggests that the crosslinking of the nanoparticles with enzyme was successful, confirming immobilization. The immobilized enzyme was further characterized and was confirmed using FTIR as shown in Figure 2C. The results showed the maximum peak area between the wavelength of 1700–2350 cm^−1^. The peak at the 2900–3000 cm^−1^ wavelength demonstrated the vibrations of C–H bond stretching and the broad band at 3430 cm^−1^ showed the stretching of the hydrogen bond in O–H groups [21]. The peaks observed at 1440–1450 cm^−1^ and below 1000 cm^−1^ indicate the vibration due to the stretching of C–O–C and C–H in the enzyme [11]. Bands observed within the area of 1560–1570 cm^−1^ depicted the bending vibrations of amide II and the stretching vibration of amide I, and a further C=O group was confirmed with the band [11,21]. The residues of C–O showed the stretching vibrations from uronic acid coming from the enzyme at 945 cm^−1^ [21]. The results obtained were significantly comparable to the study conducted for the production of oligosaccharides of alginate from *Stypocaulon scoparium* through alginate lyase [21] and where chitosan was used for immobilization of alginate lyase [11] as similar peaks were observed in these studies.

### 2.3. Enzyme Activity at Varying pH and Temperature

**Figure 3:**
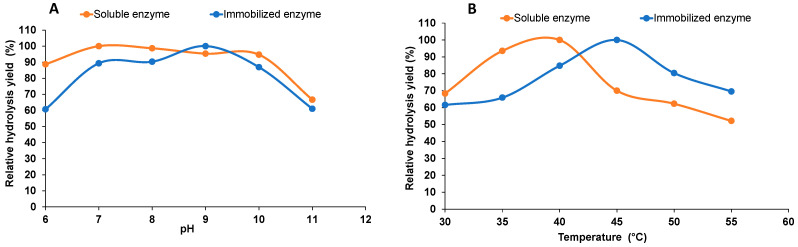
Effect of varying reaction conditions: (**A**) pH range from pH 6–11 and (**B**) temperature (30–55 °C) in the hydrolysis of sodium alginate by soluble and immobilized enzyme.

The relationship between the pH and the catalytic efficiency of the enzyme was demonstrated through the pH profile of free and immobilized alginate lyase studied in the range of pH 6 to 11 (Figure 3A). The relative enzyme activity increased for immobilized enzyme (from 60–90%) compared to free enzyme. Maximum hydrolysis of alginate by the free enzyme was 70%, achieved at pH 7, and for immobilized enzyme it was 59%, obtained at pH 9. Further increases or decreases in the pH value made the enzyme unstable due to disrupted hydrogen bonding and active sites that reduced the hydrolysis of alginate. The activity for both free and immobilized enzyme decreased above pH 9 and was observed to be 60% at pH 11 for both the enzymes. Compared to the previous studies, most of the reported enzymes were basophilic lyases and showed pH stability at higher salt concentrations (pH 8.5) [22]. Earlier studies have reported that the optimum activity of alginate lyase was at acidic pH 6 [23] and basic pH of 8 to 10 [24,25,26,27,28]. 

To investigate the effects of different temperatures, the hydrolysis of alginate by free and immobilized alginate lyase was conducted at temperatures ranging from 30 °C to 55 °C, As shown in Figure 3B the relative activity for free enzyme at 30 °C was 68.3% and for immobilized enzyme it was 61.5%. Free alginate lyase showed maximum activity from 35–40 °C, however, the immobilized alginate showed maximum activity at 45 °C. However, with the increase in temperature to 50 °C, free enzyme retained only 60% of its activity. Free enzyme showed lower activity at 45 °C while a previous study indicated that this temperature was optimum for maximum substrate degradation [20] and it was stable with maximum activity below this temperature. However, the free alginate lyase enzyme showed similar activity when compared with cold-adapted alginate lyases from *Vibrio* sp., which showed thermal stability below 35 °C [20].

Similar results in accordance with the present study were observed, which demonstrated the stability of alginate lyase immobilized on chitosan nanoparticles at 45 °C [12]. The stability of alginate lyase at 45 °C and pH 9 as compared to the free form at 37 °C and pH 7.4 might be due to the solid matrix of the nanoparticles which provides buffering capacity to the enzyme and alters the protonation pattern. Higher pH leads to enzyme–substrate complex distortion due to inactivation of enzyme, however, a higher temperature beyond the optimum point leads to the denaturation of the proteins in the enzyme [29].

### 2.4. Kinetic Study on Immobilized and Free Enzyme

The kinetic study of free and immobilized alginate lyase was carried out using varying concentrations of alginate. A Lineweaver–Burk plot was made in order to calculate the rate of reaction with respect to the substrate concentration. The value of V_max_ (maximum rate of reaction) and K_m_ (substrate concentration at which reaction rate is 50% of V_max_) was calculated according to the Lineweaver–Burk equation (Table 1). The value of V_max_ was 0.2 mg/mL/min for free and immobilized AL. K_m_ was 10.10 mM for free enzyme compared to 20 mM for the immobilized enzyme. Various reports on alginate immobilization on different supports have suggested that the shift in the K_m_ value of the enzyme is due to the immobilization which alters the substrate-binding affinity of the enzyme active sites. Previous studies have shown higher values of V_max_ of 28.99 U/mg and K_m_ of 5.5 mg/mL [8]. In another study, the value for V_max_ was calculated to be 1.6 nmol/s [30]. The values obtained from this study were lower compared to the results obtained from previous reports which could be due to the different origin and source of the alginate lyase.

### 2.5. Thermal Stability and Storage Stability of Alginate Lyase

**Figure 4:**
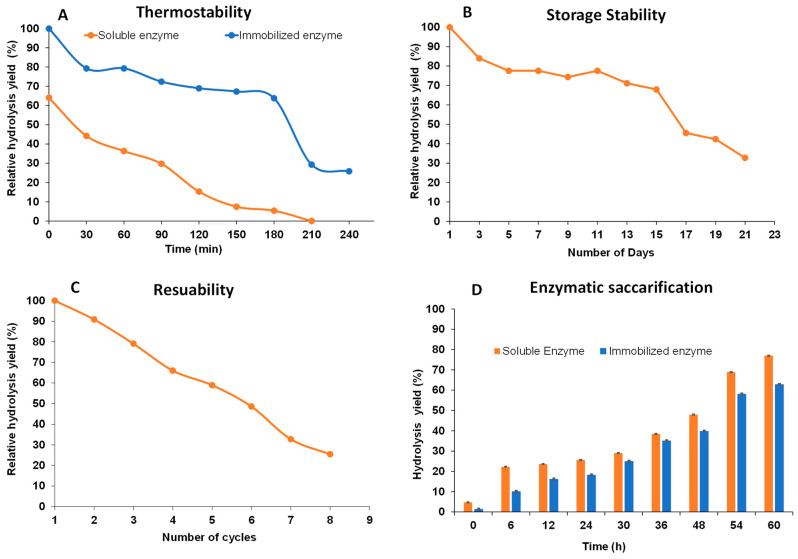
(**A**) Thermostability studies of soluble and immobilized enzyme (45 °C); (**B**) Reusability studies of immobilized alginate lyase after storage of immobilized alginate lyase (every third day when stored at 4 °C) for 23 consecutive days; (**C**) Activity of immobilized enzyme was determined for 8 consecutive cycles; and (**D**) Hydrolysis of alginate sourced from a raw seaweed (bull kelp) biomass by free and immobilized enzyme for 60 h.

The effect of immobilization on the thermal stability of the enzyme was determined by incubating the enzyme at 45 °C for different time intervals of 30, 60, 90, 120, 150, and 180 min and calculating its activity by conducting hydrolysis. The immobilized alginate lyase retained 79% of its activity when kept at 45 °C for 60 min as shown in Figure 4A. However, free enzyme retained 64% activity which is lower in comparison to the immobilized enzyme. There was a significant reduction in the activity of free enzyme from 60 min to 90 min, however, the activity of immobilized alginate lyase was reduced only by 8% after 90 min of incubation at 45 °C. Free enzyme retained only 29% activity after 2 h of incubation, however, immobilized enzyme retained 69% of its original activity. Constant reduction in the activity of free enzyme was observed as it was reduced to 15.2 ± 0.01% in 2.5 h and 7.4 ± 0.01% after 3 h, whereas the immobilized enzyme retained 62% of its activity for up to 3 h. An increase in the temperature caused the denaturation of the enzyme and resulted in reduced activity though results indicated a negligible effect of elevated temperature on the properties of the enzyme after immobilization, with the exception of higher stability at higher temperatures. As compared to previous reports, immobilized enzyme retained 76% of its activity when incubated for 1 h at 45 °C with magnetic nanoparticles and tannic acid [8]. Another study demonstrated 83.7% activity of the enzyme immobilized on mesoporous titanium oxide particles when incubated at the same temperature and for the same period of time [1]. In addition to improved thermostability, results demonstrated improvement in the storage stability of the immobilized enzyme. Immobilized alginate lyase showed 84% activity after 3 days of storage at 4 °C. The relative activity was reduced to 78% on day 5 and remained stable until day 11. Further, the relative activity of immobilized alginate lyase was found to be ~50% until day 16 at 4 °C storage and showed stability (32.7% of relative activity) up to 21 days (Figure 4B). For the alginate lyase (AlgL17 from recombinant *E. coli*) immobilized on Fe_3_O_4_, nearly 60% (incubated at 4 °C) of relative activity was retained after 21 days [8].

### 2.6. Reusability of Immobilized Enzyme

The reusability of the immobilized alginate lyase was analyzed by reusing the conjugate mix for eight consecutive cycles. The magnetic nature of the nanoparticles facilitated the separation and reuse of the same enzyme for consecutive cycles which makes it a cost-efficient process like other direct degradation enzymatic hydrolysis methods [31]. Immobilized alginate lyase retained 91% of its activity when it was reused for a second cycle. It showed ~80%, 66%, and 60% activity during the 3rd, 4th, and 5th cycles, suggesting a gradual decrease in its activity. The activity of immobilized enzyme was reduced to 25% when reused for an 8th cycle as shown in Figure 4C. Our results agreed with Jiang et al.’s [25] who report the use of Fe_3_O_4_ nanoparticles for immobilized alginate lyase where immobilized enzyme exhibited 70% activity till the 5th cycle and it declined to one third residual activity (after 25 days) [8]. Other reports also demonstrated that the activity of the immobilized enzyme (using low-molecular-weight chitosan nanoparticles) was maintained at 60% by the end of the 6th cycle [12] and about 66% of the relative activity was observed after using it for seven cycles with an alginate lyase from *Arthrobacter* sp. AD10 [29].

### 2.7. Enzymatic Saccharification of Raw Seaweed Biomass

The immobilized and free enzymes were used to hydrolyze raw seaweed biomass and 77% hydrolysis of seaweed biomass was shown by free enzyme in 60 h, however, the immobilized enzyme showed 63% hydrolysis in 60 h (Figure 4D). Previous studies indicated 60.5% hydrolysis of the biomass in 12 h when alginate lyase was isolated from microbes (*Pseudoalteromonas* sp.) [32]. The present study demonstrated improvement in the hydrolysis of biomass by using immobilized alginate lyase.

### 2.8. High-Performance Liquid Chromatography (HPLC) Analysis

The HPLC profile exhibited similar monosaccharide yields of 20.5% with free enzyme and 17.6% with immobilized alginate lyase. The investigation showed that the sugar hydrolysate contained variable quantities of monosaccharide sugars such as guluronic acid, mannuronic acid, mannose, galactose, xylose, and fucose as shown in Table 2. However, the glucose level was significantly higher for alginate hydrolyzed by the free enzyme than the immobilized enzyme. Immobilized AL released 3% less monosaccharide compared to hydrolysis by free enzyme. No previous reports were found in the literature similar to this study which endorsed the novelty of the current research work. The molecular weight profile of the oligosaccharides during the hydrolysis of seaweed biomass suggests that with the increase in the incubation time the molecular weight of the released oligosaccharides decreases relatively more for the immobilized AL than the free enzyme. This could be due to the enhanced biochemical properties of the immobilized enzyme; the hydrolysis has been improved. However, the hydrolysis of biomass with the free enzyme led to the production of oligosaccharides with higher molecular weight ranging from 937 to 845 kDa compared to the immobilized enzyme which had a broader range from 1040 to 371 over 60 h of incubation. The hydrolysate obtained from 30–60 h from the immobilized enzyme contained 60% of the oligosaccharides with a molecular weight of 370–470 kDa and about 2–5% of hydrolysate was 1–6 kDa. However, hydrolysate produced by the free enzyme contained ~30% of all the fractions with molecular weight of 6 kDa and nearly 17–20% of fractions were below 6 kDa (data provided in Supplementary Materials in Figures S1–S5). Based on a previous study [33], alginate oligosaccharides (AOSs) with low molecular weight (MW) of 414–865 kDa promote cellular proliferation, those of 414–1602 kDa promote immunomodulatory activity, ~5000 Da AOSs enhance hypoglycemic activity, and ~1500 Da AOSs enhance antioxidant activity.

### 2.9. Antioxidant Assay

According to the study conducted by Jiang et al. [25], increasing the alginate oligosaccharide (AOS) concentration contributes to stimulating the antioxidant defense in cells through enzymes including superoxide dismutase (SOD), catalase (CAT), and glutathione (GSH) [8]. The effect was nearly double (with SOD and GSH) or more than double (with CAT) when the concentration of AOS was increased by 8 times. However, in the present study, the ferric-reducing antioxidant power was 0.13 mM FeSO_4_ for the free enzyme and it increased with the hydrolysis time. For the immobilized enzyme, ferric-reducing antioxidant power was 0.16 mM at 0 h and it increased by more than double—0.37 mM FeSO_4_—as the hydrolysis of biomass reached 60 h. The antioxidant activity shown by immobilized-alginate-lyase-catalyzed hydrolysis was significantly higher than the soluble enzyme-based hydrolysis which could be due to the presence of magnetic nanoparticles with iron molecules. 

## 3. Materials and Methods

All the chemicals used in this study, including alginate lyase (*Flavobacterium sphingomonas*, 10,000 U/g, catalogue no. A1603-100MG), sodium alginate, trizma^®^ base, protein assay kit (Folin and Ciocalteu’s phenol reagent), 5-dinitrosalicylic acid (DNS), calcium chloride, cobalt bromide, copper sulfate, glucose, iron chloride, magnesium sulfate, manganese chloride, monopotassium phosphate, potassium sodium tartrate tetrahydrate, sodium chloride, sodium hydroxide, zinc sulfate, and nanoparticles (iron oxide 8365W), were procured from Sigma Aldrich Pty. Ltd. (St. Louis, MO, USA) and glutaraldehyde from Merck, Germany.

### 3.1. Enzyme Assay

The enzyme activity of free and immobilized AL was determined using the DNS method [34,35]. The assay for the free enzyme was conducted at 37 °C with a reaction mixture containing 25 IU of enzyme (alginate lyase—100 µL) and 1 mL of 1.5%, *w*/*v* of alginate dissolved in 20 mM tris buffer (pH 7.0) and incubated for 2 h in a water bath (Ratek Instruments Pty. Ltd., Boronia, VIC, Australia). The reaction was stopped by adding 1 mL of DNS reagent and boiled for 10 min in a vigorously boiling water bath and immediately cooled in an ice bath for 5 min. The concentrations of total reducing sugar released were measured at 540 nm using an Omega plate reader (Ω) (FLUOstar Omega, BMG Labtech, Mornington, VIC, Australia). One unit of enzyme activity is defined as 1 μM of glucose liberated per minute [32].

Enzyme Activity (IU/mL) = Reducing sugar released (product) (µmol/mL)/Time (min)

### 3.2. Effect of Different Metal Ions on Enzymatic Activity

To determine the effect of different metal ions (monovalent, divalent, and trivalent) on the activity of alginate lyase, 25 IU of enzyme (50 µL in Tris buffer) was incubated with 1 mL of substrate (50 µL, 1 mM) and metal ions including K^+^, Na^+^, Mn^2+^, Zn^2+^, Ca^2+^, Cu^2+^, Mg^2+^, Co^3+^, and Fe^3+^, SDS, and EDTA for 2 h [36]. The DNS assay was performed to analyze the effect of these ions on enzyme activity.

### 3.3. Immobilization of Alginate Lyase

Magnetic nanoparticles (NPs) were used for the immobilization of alginate lyase (AL). The nanoparticles were first suspended in Milli-Q water and then sonicated (SONICS Vibra cell^TM^, Newtown, CT, USA) for 30 min to homogenize the particles and to achieve a uniform size [35]. Glutaraldehyde was used as a crosslinker between NPs and AL. To achieve an efficient binding between NPs and crosslinker, 1 mL of glutaraldehyde was mixed with 1 mL of NPs at a concentration of 5 mg/mL in Milli-Q water and incubated for different time intervals (30, 60, 90, 120, and 180 min) [14]. Before immobilization, the activated NPs were washed twice with the Milli-Q water and once with the Tris buffer (20 mM, pH 7.4). The immobilization was conducted by incubating AL and activated NPs in a water bath for 2 h at 37 °C. The binding efficiency of AL was determined by protein loaded onto the NPs using the Lowry method by incubating different enzyme units (1, 10, 50, 100, 150, 200, and 250 IU) with 5 mg of nanoparticles. The unbound protein was removed by washing the immobilized NP conjugate twice with water and once with Tris buffer [15]. To determine the binding efficiency, the supernatant from the washing step was used for the calculation of unbound protein using the Lowry method [37]. The protein-binding efficiency was determined using the following formula [35]:

$$\mathrm{Binding}\;\mathrm{efficiency}\;\left(\%\right)=\frac{\mathrm{Total}\;\mathrm{amount}\;\mathrm{of}\;\mathrm{protein}\;\mathrm{bound}\times 100}{\mathrm{Total}\;\mathrm{amount}\;\mathrm{of}\;\mathrm{protein}\;\mathrm{added}}$$

### 3.4. Characterization of Immobilized Alginate Lyase

The morphology and distribution of AL onto nanoparticles were determined by a scanning electron microscope (FEI INSPECT F50, Bath, UK). The samples were mounted on an aluminum stub, sputtered with 20 nm thick platinum, and observed under the SEM at an accelerating voltage of 5 kV [35]. Fourier transform infrared (FTIR) spectroscopy was performed to determine the binding between NPs and AL using a Shimadzu infrared spectrophotometer (Nicolet Nexus 870 step-scan FTIR) with diamond crystal to derive the spectra. Samples were scanned over the wavenumber range of 400–4000 cm^−1^ with a 32-scan resolution and the spectra were analyzed [11]. 

### 3.5. Temperature and pH Optimization

The optimum temperature for the free and immobilized AL was determined by incubating 100 μL of enzyme (25 IU) with 1 mL of 1.5% *w*/*v* of alginate at different temperatures—30, 35, 40, 45, 50, and 55 °C—in a water bath for 2 h. The enzyme activity was determined using the DNS method and reducing sugar was estimated at 540 nm [38]. To determine the optimum pH value, free and immobilized enzymes were incubated at different pH—6, 7, 8, 9, 10, and 11—using Tris buffer (20 mM). All experiments were conducted in triplicate and results reported as mean values ± standard deviation.

### 3.6. Determination of Enzyme Kinetics

The kinetic study of free and immobilized alginate lyase was conducted by incubating alginate at different concentrations—0.5%, 1%, 1.5%, 2%, 2.5%, 3%, 3.5%, and 4% *w*/*v*—using 25 IU of free (37 °C) and immobilized (45 °C) enzyme. The reaction rate was determined by the substrate being utilized or the total reducing sugar released per unit time [29,39]. The kinetics of the enzyme was derived by calculating the K_m_ and V_max_ values by plotting a Lineweaver–Burk plot [29].

### 3.7. Thermostability and Storage Stability

To determine the thermal stability, immobilized AL was incubated at 45 °C for different time intervals—30, 60, 90, 120, 150, and 180 min—without substrate (alginate) and then tested for hydrolysis with alginate (1.5% *w*/*v*). To determine the half-life of the enzyme, the immobilized enzyme was tested until reaching 50% of its activity [8]. For the storage study, the immobilized enzyme was incubated at 4 °C in the refrigerator for 30 days and the activity was measured using 1.5% (*w*/*v*) alginate after 1, 3, 5, 7, and 21 days using the DNS assay [8].

### 3.8. Reusability

To study the reusability of immobilized AL, substrate hydrolysis was conducted at 45 °C. The enzyme was washed with Milli-Q water and then resuspended in Tris buffer, which was reused for the following cycle. The activity calculated for the first cycle was considered as 100% (control) and the hydrolysis cycle was repeated until the activity declined to below 50% [35]. 

### 3.9. Enzyme Saccharification of Seaweed Biomass

Enzymatic hydrolysis of the dried raw biomass (0.5%, *w*/*v* of bull kelp, 250 μm) was performed using free alginate lyase at 37 °C and immobilized alginate lyase at 45 °C for 60 h [40]. The release of total reducing sugars was determined at an interval of every 6 h for the following timepoints: 0, 6, 12, 24, 30, 36, 48, 54, and 60 h [34,35]. The hydrolysis of raw biomass was calculated using the following formula [35]:

$$\mathrm{Alginate}\;\mathrm{hydrolysis}\;=\frac{\mathrm{Reducing}\;\mathrm{sugar}\;\mathrm{released}\;\left(\mathrm{mg}\right)\times 0.9\;\mathrm{correction}\;\mathrm{factor}\;\times 100}{\mathrm{Substrate}\;\left(\mathrm{mg}\right)}$$

### 3.10. Antioxidant Assay

The antioxidant activity was determined using the FRAP assay. The assay was performed by adding 180 µL FRAP reagent (25 mL acetate buffer + 2.5 mL TPTZ solution + 2.5 mL FeCl_3_.6H O) to 24 µL of samples (hydrolysate obtained at intervals of 6 h while conducting hydrolysis of biomass) and vortexed. The blank was 24 µL of distilled water and 180 µL of FRAP reagent [41]. After 10 min of incubation in the dark and at room temperature, absorbance at 593 nm was measured using a plate reader (Fluostar Omega, BMG Labtech, Mornington, VIC, Australia). The control sample was a mixture of 3 mL of FRAP reagent and 1 mL of distilled water. The results were calculated according to a calibration curve and expressed as µM Fe^2+^/mL [42]. 

### 3.11. High-Performance Liquid Chromatography Analysis

The reducing sugar released from the raw seaweed biomass hydrolysate was quantified using high-performance liquid chromatography (HPLC-UFLC XR SHIMADZU, Mundelein, IL, USA) and with a Phenomenex column (Kinetex C18, 2.6 μm, 3 × 100 mm 100A). HPLC was equipped with a solvent degasser, quaternary pump, auto-sampler, thermostat column compartment, and a refractive index detector [43]. Monosaccharide sugars including glucose, cellobiose, arabinose, mannose, and xylose and organic acids including succinic acid, oxalic acid, acetic acid, formic acid, and ethanol were procured from Sigma, St. Louis, MO, USA (HPLC grade) and used as the standards. 2-deoxy glucose (2-DOG) was used as an internal standard and the concentration of each monosaccharide was estimated by extrapolating the standard curve. All the chemicals used were of standard analytical grades and the run was performed in four replicates and represented with standard deviation (±SD) [44]. The molecular weight distribution of alginate oligosaccharides was determined using size-exclusion chromatography [29]. The size separation was conducted using PolySep GFC-P5000 and PolySep-GFC-P6000 columns (Phenomenex, Torrance, CA, USA) in a Prominence UHPLC (Shimadzu-LC20A XR system, Tokyo, Japan) equipped with refractive index detector (RID-10A, Shimadzu, Kyoto, Japan). The samples were diluted using 0.1 M sodium nitrate in 1:2 ratio (sample:sodium nitrate) and injected into the system. Dextran with different molecular weights—65, 195, 400, and 1050 kDa—was used as the standard. Sodium nitrate (0.1 M) was used as the mobile phase at a flow rate of 1 mL/min. All the chemicals used in this study were of HPLC analytical grade and procured from Sigma-Aldrich. (St. Louis, MO, USA) [43].

## 4. Conclusions

In the current study, the alginate lyase was immobilized onto a nanocarrier with magnetic properties to facilitate its separation from the reaction mixture and reuse. The successful hydrolysis of alginate was achieved with more than 75% hydrolysis with the free enzyme under an optimized enzyme concentration, pH, and temperature along with the effect of metal ions and kinetic study. Results obtained for the immobilized enzyme confirmed the activity and thermal stability for 3 h at a higher temperature. Furthermore, immobilization enhanced the stability of the enzyme at an elevated pH from 7 to 9 with reusability for six consecutive cycles with 50% of its activity. Immobilized alginate lyase activity can be retained by storing it at 4 °C for more than 30 days. Upon hydrolyzing seaweed biomass using free and immobilized enzyme, no significant variation was observed in the monosaccharide yield between free and immobilized enzyme. However, a significant variation was observed in the hydrolysis pattern with the immobilized enzyme, and the maximum fraction of hydrolysate was composed of oligosaccharides with higher molecular weight—60% 370–470 kDa—as compared to the free enzyme with 30% 6 kDa. This study demonstrated the long-term reusability and storage of immobilized enzyme which suggests it is a cost-effective and environmentally sustainable approach for the bioprocessing of seaweed to fulfil the industrial demand.

## Figures and Tables

**Table 1:** The effect of immobilization on the affinity of enzymes (K_m_) and rate of reaction compared to soluble enzyme.

Parameters	Soluble Enzyme	Immobilized Enzyme
K_m_ (mM)	10.10	20
V_max_ (mg/mL/min)	0.2	0.2

Where K_m_ = Michaelis–Menten constant, V_max_ = maximum rate of reaction.

**Table 2:** The total reducing sugar released from seaweed after 60 h of hydrolysis by soluble and immobilized enzyme.

Oligosaccharides	Soluble Enzyme (%, *w*/*w*)	Immobilized Enzyme (%, *w*/*w*)
Guluronic Acid	1.20	1.62
Mannuronic Acid	10.25	9.80
Mannose	0.95	0.73
Ribose	0.17	0.00
Rhamnose	0.26	0.22
Glucuronic Acid	0.38	0.51
Galacturonic Acid	0.14	0.27
Glucose	3.63	1.89
Galactose	0.72	0.49
Xylose	0.56	0.41
Arabinose	0.25	0.05
Fucose	2.01	1.70
Total	20.52	17.68

## Data Availability

The original data presented in the study are included in the article/Supplementary Material; further inquiries can be directed to the corresponding author.

## Supplementary Materials

The following supporting information can be downloaded at: https://www.mdpi.com/article/10.3390/md22030120/s1, Figure S1: Effect of increasing concentration of enzyme units (International units: IU) on the hydrolysis yield of alginate by alginate lyase; Figure S2: Effect of increasing substrate concentration on the hydrolysis yield of alginate by alginate lyase; Figure S3: Effect of temperature (a: wide range, b: narrow range) on the hydrolysis yield of alginate by alginate lyase; Figure S4: Effect of pH (a: wide range, b: narrow range) on the hydrolysis yield of alginate by alginate lyase; Figure S5: Effect of shaking and time of incubation on the hydrolysis yield of alginate by alginate lyase.
