# Supplementary material for: Production of Alginate Oligosaccharides (AOSs) Using Enhanced Physicochemical Properties of Immobilized Alginate Lyase for Industrial Application
Source: Mar Drugs. 2024 Mar 4;22(3):120. doi: 10.3390/md22030120 (PMC10971502; doi:10.3390/md22030120)
Supplement: Supplementary file 1 [file marinedrugs-22-00120-s001.zip › marinedrugs-2766319-supplementary.pdf]

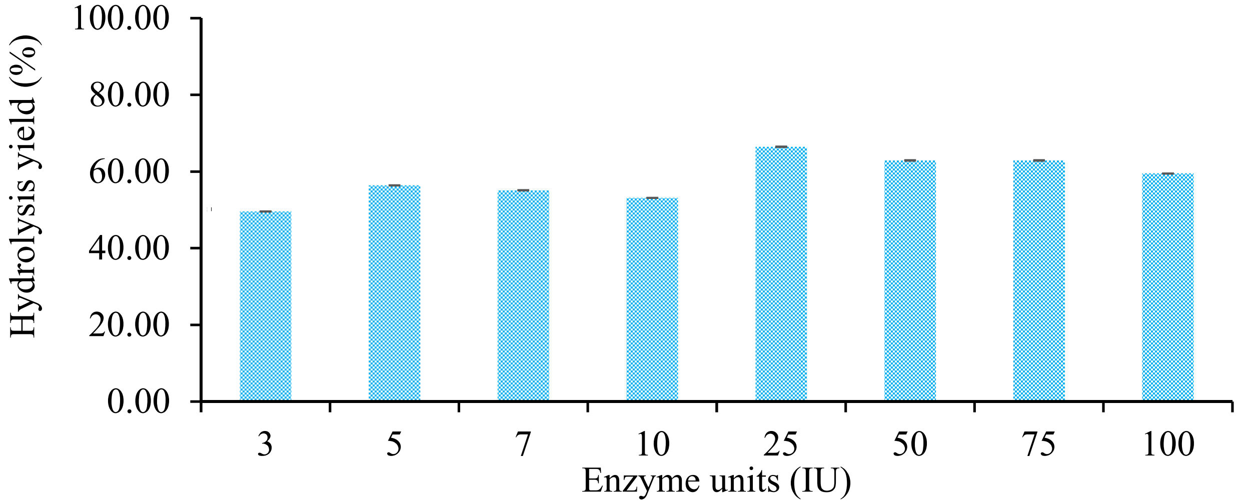

Figure S1. Effect of increasing concentration of enzyme units (International units: IU) on the hydrolysis yield of alginate by alginate lyase

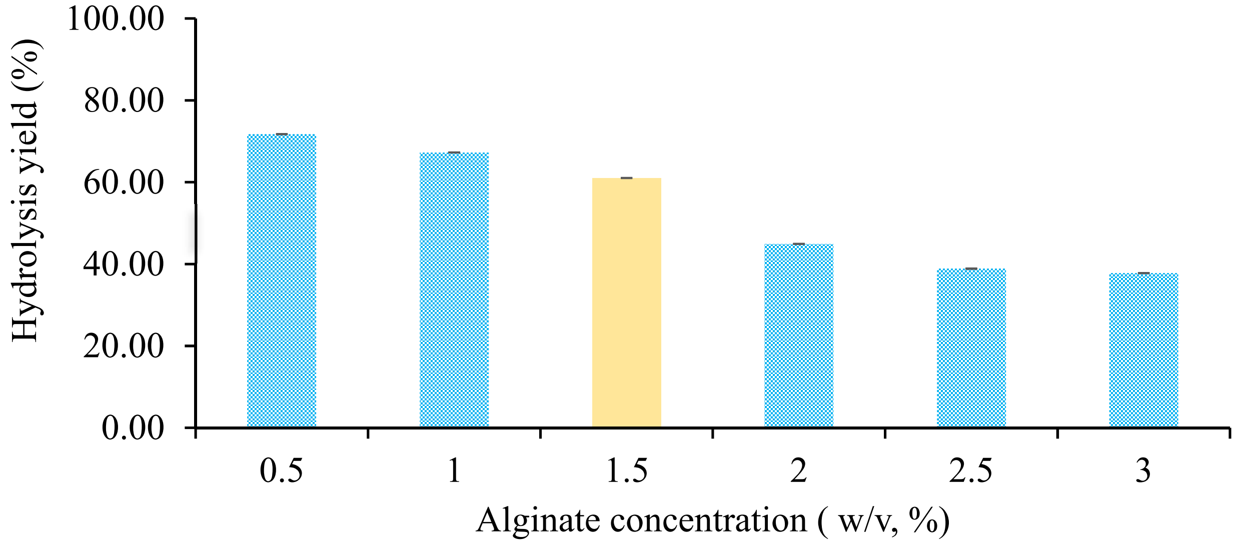

Figure S2. Effect of increasing substrate concentration on the hydrolysis yield of alginate by alginate lyase

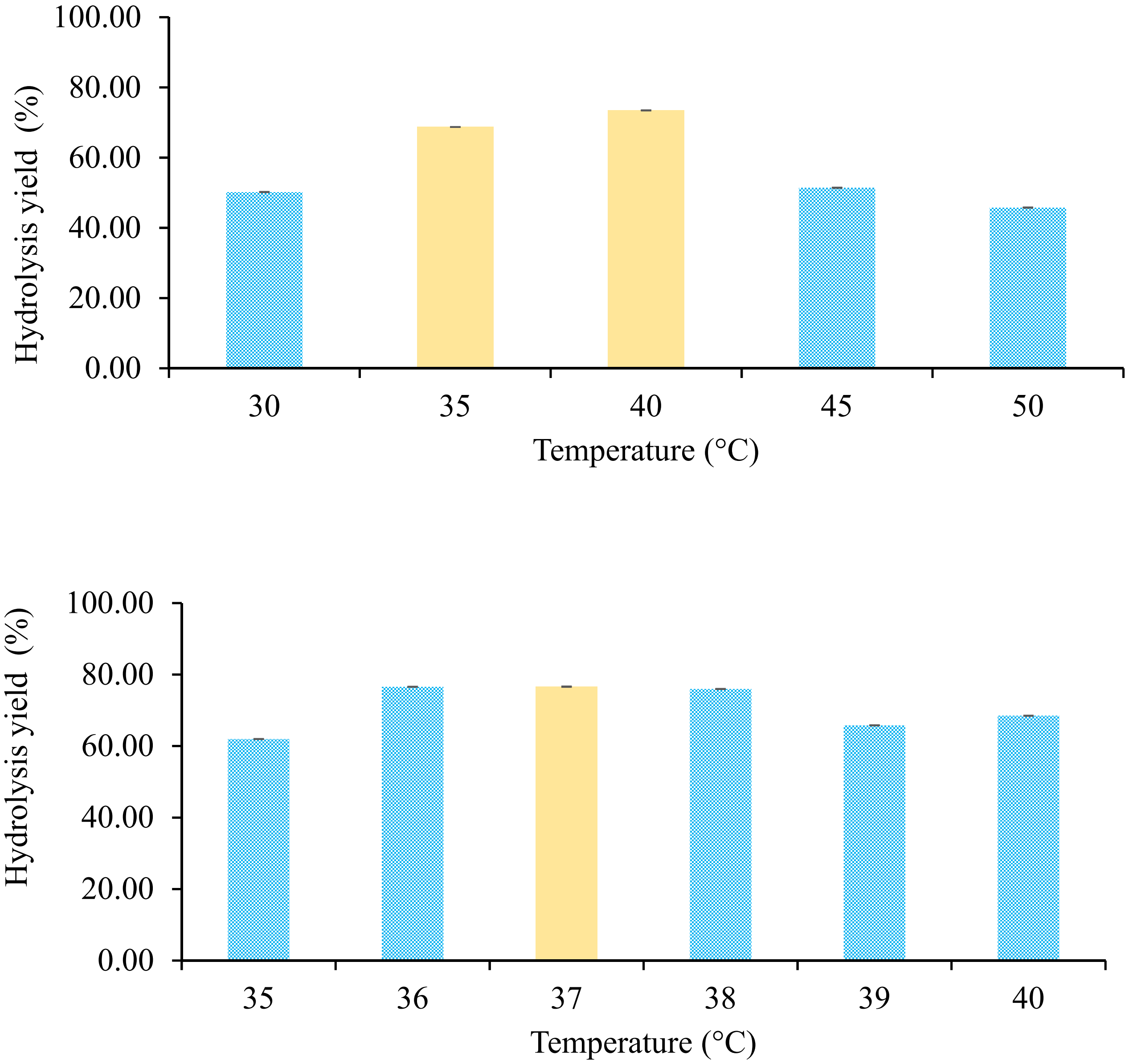

Figure S3. Effect of temperature (a: wide range, b: narrow range) on the hydrolysis yield of alginate by alginate lyase

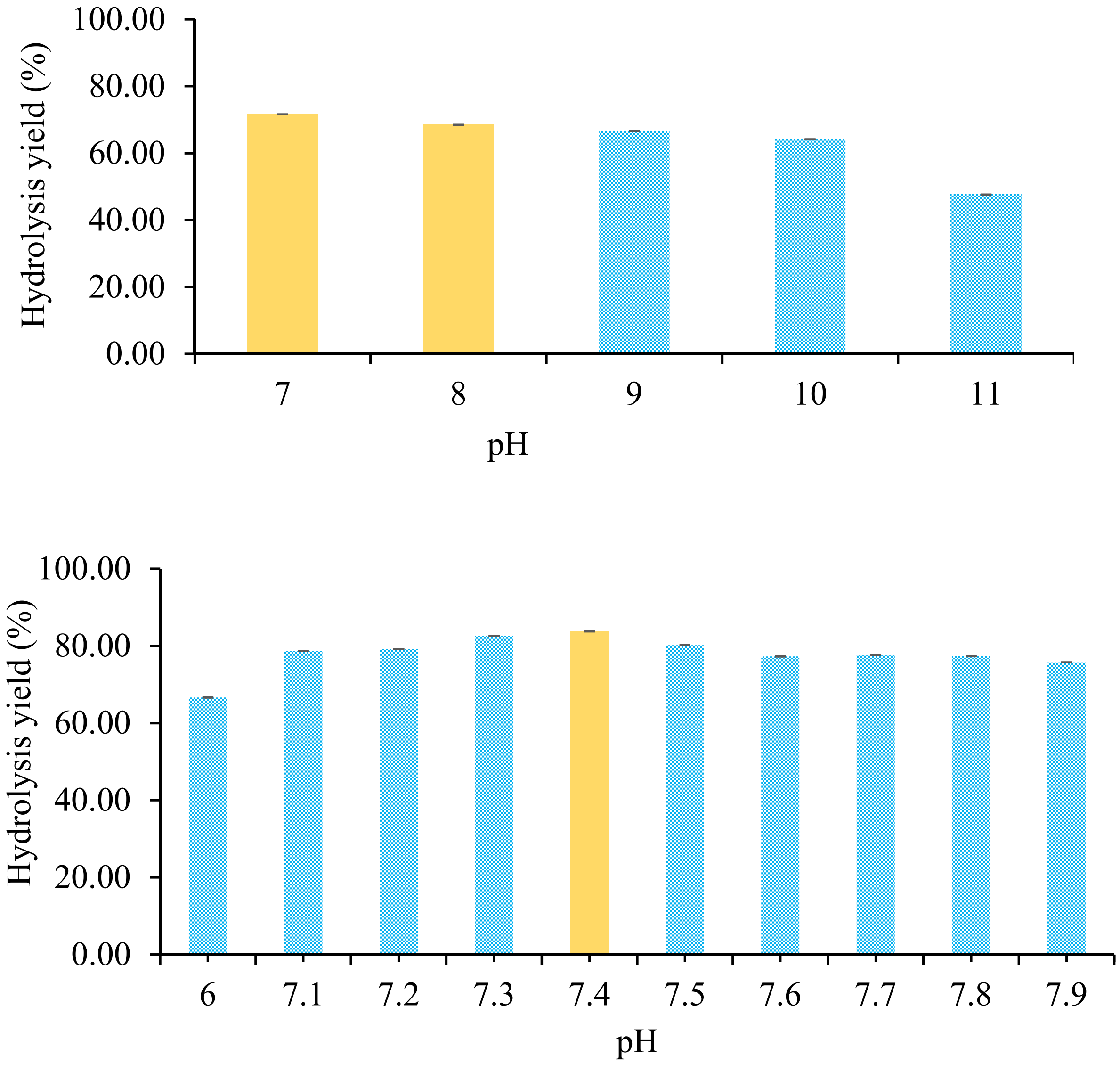

Figure S4. Effect of pH (a: wide range, b: narrow range) on the hydrolysis yield of alginate by alginate lyase

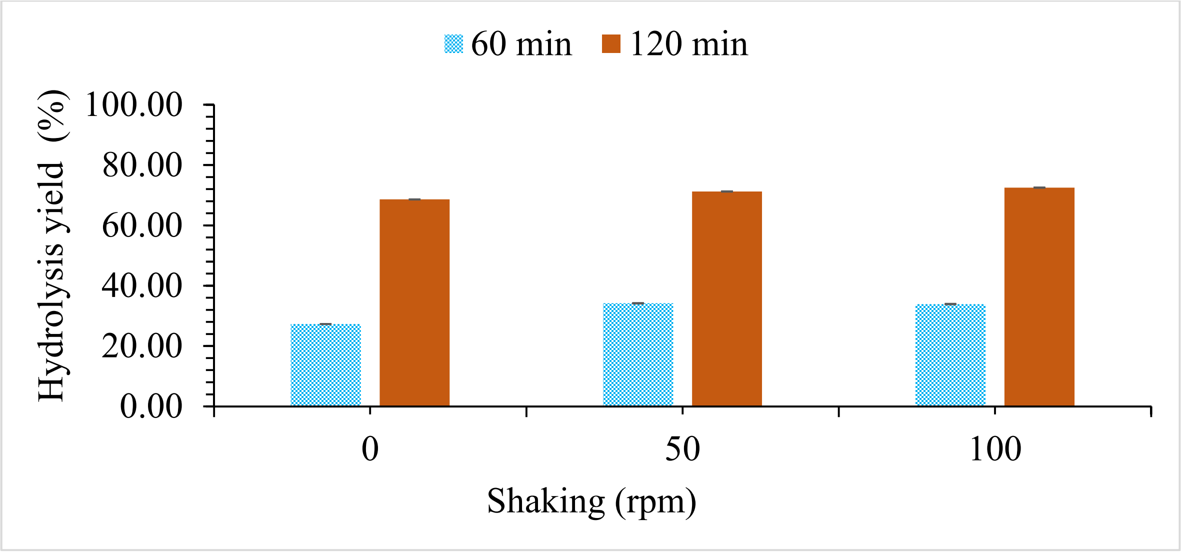

Figure S5. Effect of shaking and time of incubation on the hydrolysis yield of alginate by alginate lyase
